# Long-Term GPS Tracking of Ocean Sunfish *Mola mola* Offers a New Direction in Fish Monitoring

**DOI:** 10.1371/journal.pone.0007351

**Published:** 2009-10-09

**Authors:** David W. Sims, Nuno Queiroz, Nicolas E. Humphries, Fernando P. Lima, Graeme C. Hays

**Affiliations:** 1 Marine Biological Association of the United Kingdom, The Laboratory, Plymouth, United Kingdom; 2 School of Biological Sciences, University of Plymouth, Plymouth, United Kingdom; 3 CIBIO – Universidade de Porto, Campus Agrário de Vairão, Vairão, Portugal; 4 Department of Biological Sciences, University of South Carolina, Columbia, South Carolina, United States of America; 5 Institute of Environmental Sustainability, Swansea University, Swansea, United Kingdom; Institut Pluridisciplinaire Hubert Curien, France

## Abstract

Satellite tracking of large pelagic fish provides insights on free-ranging behaviour, distributions and population structuring. Up to now, such fish have been tracked remotely using two principal methods: direct positioning of transmitters by Argos polar-orbiting satellites, and satellite relay of tag-derived light-level data for *post hoc* track reconstruction. Error fields associated with positions determined by these methods range from hundreds of metres to hundreds of kilometres. However, low spatial accuracy of tracks masks important details, such as foraging patterns. Here we use a fast-acquisition global positioning system (Fastloc GPS) tag with remote data retrieval to track long-term movements, in near real time and position accuracy of <70 m, of the world's largest bony fish, the ocean sunfish *Mola mola*. Search-like movements occurred over at least three distinct spatial scales. At fine scales, sunfish spent longer in highly localised areas with faster, straighter excursions between them. These ‘stopovers’ during long-distance movement appear consistent with finding and exploiting food patches. This demonstrates the feasibility of GPS tagging to provide tracks of unparalleled accuracy for monitoring movements of large pelagic fish, and with nearly four times as many locations obtained by the GPS tag than by a conventional Argos transmitter. The results signal the potential of GPS-tagged pelagic fish that surface regularly to be detectors of resource ‘hotspots’ in the blue ocean and provides a new capability for understanding large pelagic fish behaviour and habitat use that is relevant to ocean management and species conservation.

## Introduction

Determining the movements of individual free-ranging animals is important for a number of reasons such as assessing patterns of habitat utilisation, prey search strategies and defining critical conservation areas. Technological developments have transformed our ability to track a broad range of animals [1], [2]. For example, Global Positioning System (GPS) tracking has been widely employed with terrestrial animals and birds, e.g. [3], and more recently with diving marine species, such as turtles and seals, using new systems that allow very rapid acquisition of GPS precision positional data (ephemeris) when individuals surface to breathe, e.g. [4]. However, long-term GPS tracking of fish has remained elusive despite huge interest in describing fish movements [5].

The movements and behaviour of large pelagic fish such as sharks and tuna have been tracked over large spatio-temporal scales by remote means using two main approaches: (i) direct, near real time positioning of animal-attached platform terminal transmitters (PTTs) by Argos polar-orbiting satellites, and (ii) satellite relay of tag-derived light-level data for *post hoc* track reconstruction [6]. These techniques have provided great insights into fish migratory movements [7], foraging patterns [8] and population structuring [9], for example. Nonetheless, since those first studies demonstrating the utility of Argos [10] and light-level geolocation [11], [12] tracking methods for fish, field validations have reported positional errors of no better than hundreds of metres for Argos tracking [13] and up to hundreds of kilometres for light-level geolocation [14]. Spatial errors of this magnitude for the comparatively low daily movement distances recorded for tracked fish (when compared to seabirds for instance) have shown that erroneous detections of particular behaviour types associated with searching and foraging are possible [15]. It is all the more surprising therefore that, to our knowledge, more highly spatially resolved tracking methodologies such as GPS have not been employed hitherto for large open-ocean pelagic fish, even though this would likely improve considerably our understanding of their behaviour during migrations, habitat selection, and when foraging.

In this paper we describe the first long-term GPS tracking of a large pelagic fish that surfaces relatively frequently [16], the ocean sunfish *Mola mola*, and which sets the scene for a new era in fish biotelemetry.

## Materials and Methods

**Figure 1:**
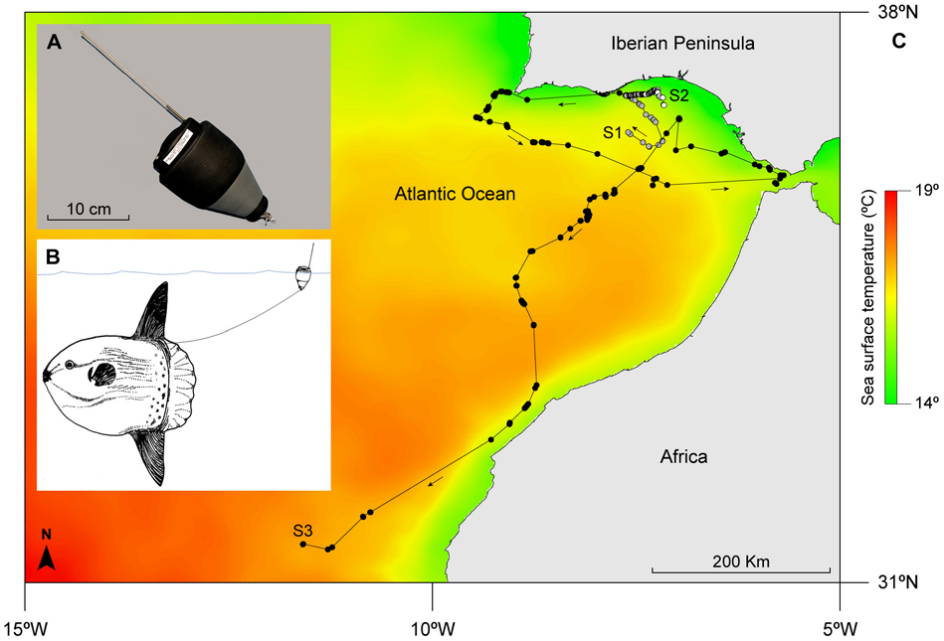
GPS tracks of ocean sunfish. (A) The fast-acquisition (Fastloc) GPS tag (depth rated to 1000 m) used to track sunfish in the north-east Atlantic (attachment method shown in B). (C) Three Tracks (S1–3) overlaid on a high resolution resolution (2 km) SST map averaged for the period between 6 November 2008 and 6 February 2009 (corresponding to track duration of S3).

Three ocean sunfish *Mola mola* (numbered S1–3, with total lengths of 0.6, 0.6 and 1.0 m, respectively) captured in a tuna pound net off southern Portugal [16] (Table 1) were each fitted with an integrated Fastloc GPS receiver and Argos Platform Terminal Transmitter (PTT) (Sirtrack Ltd, Havelock North, New Zealand) mounted in a cylindrical housing with a wrap-around buoyant ‘collar’ and hydrodynamic cone to reduce drag (overall tag height, 150 mm; float width, 80 mm; Argos antenna length, 171 mm) (Figure 1A). The tag was towed behind the fish via a 1.5-m long monofilament tether that was attached to the fish's dorsal surface with a T-bar anchor tag (Figure 1B). This tether length was chosen as a trade-off between the need for the tag to have a good chance of breaking the sea surface to transmit in air when the sunfish was near the surface, and the need to minimise drag to the fish and any interference of the tag with fin movements during swimming. With this tether length, the tag floated clear of the fish and above and behind the dorsum (see Figure 1B) and although the attachment likely increased drag, it did not interfere with fin movements or continually bump the dorsal surface. This species is known to dive to at least 472 m depth and can often remain at deep depths for long periods only returning occasionally, and then often only briefly to the surface [16]. In the light of this behaviour, the number of transmissions per day achieved with our attachment method onto sunfish supported our choice of tether length as perhaps a reasonable trade-off between transmission likelihood and drag-induced compromises to sunfish swimming. A saltwater switch located near the Argos antenna conserved battery power when the tag was submerged, however when dry at the surface in air the Fastloc receiver was set to acquire the GPS position every 45 s, with subsequent Argos transmission of messages containing the encoded GPS data every 60 s.

**Table 1:** Summary of GPS tag deployments on ocean sunfish *Mola mola*.

Fish #	Total length (m)	Date captured	Capture location	Final location	Track days	Distance travelled (km)
1	0.6	14 May 2008	37.02°N 7.71°W	36.53°N 7.60°W	15	150.5
2	0.6	14 May 2008	37.02°N 7.71°W	36.86°N 7.16°W	5	98.1
3	1.0	6 Nov 2008	37.02°N 7.71°W	31.47°N 11.59°W	92	1818.6

Two time series of locations were retrieved remotely from tags towed by sunfish. The first comprised standard Argos locations determined from the Doppler shift in the receiver-uplink signal frequency as the satellite passes overhead [reported accuracy: service providers, 150 m (LC3) to >1000 m (LC0); field tests (mean ± S.D.), 482 m ±153 (LC3) to 5179 m ±3677 (LC0)] [13]. The second time series was calculated from remotely retrieved GPS data decoded from Argos messages by post-processing performed by K. Lay (Sirtrack Ltd) using the manufacturer's proprietary software (data available: date, time, latitude, longitude, number of satellites used). Fastloc GPS locations were plotted using ArcGIS and filtered for positional errors, firstly removing swimming speeds above 3 m s^−1^, then by reference to the number of satellites acquired to resolve each location. Because Fastloc GPS location estimates vary, with a higher number of satellites generally yielding more accurate locations, we removed positions with <5 satellites [reported field-test spatial errors (mean ± S.D.): 8 satellites, 26 m ±19.2; 5 satellites, 64 m ±79.4] [13].

The time series of filtered GPS locations were each analysed in ArcGIS to determine distances and times between consecutive positions, thus giving over-the-ground speed estimates. Paths were then mapped on time-referenced, remotely-sensed images of sea surface temperature (SST) (http://www.medspiration.org/), sea surface height (SSH) and geostrophic currents (http://www.aviso.oceanobs.com/; http://www.ocean.nrlssc.navy.mil/global_nlom/) (direction and speed vectors) (see Figure legends for more details). To examine changes in movement path tortuosity as a function of spatial scale, the first passage times (FPT) were calculated using custom-written software (Track Analysis v.4, Marine Biological Association, 2009). FPT is defined as the time required for an animal to cross a circle of given radius [15]. To calculate the first passage times along a path, a circle of smallest given radius was moved along the path at equidistant points by creating intermittent steps along the tracked path, with this procedure repeated for circles of increasing radius. From these iterations, the estimated relative variance, 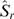, in FPT is calculated as a function of *r*: 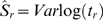 where *t_r_* is the FPT for a circle of radius *r* [15].

## Results

A total of 612 Fastloc GPS locations were retrieved remotely from tags via Argos, with 3.0, 44.2 and 3.8 locations d^−1^ for sunfish 1, 2 and 3 respectively. The highest number of locations per day (221 positions in 5 days) was obtained for sunfish 2 presumably because this individual spent more time shallower than 1.5 m compared with the two other tagged sunfish. For the longest track (S3), 346 GPS locations were obtained, with 67.1% of GPS positions fixed from between 5 and 8 satellites, compared with only 91 conventional Argos PTT locations of lower spatial accuracy for S3.

Sunfish 1 and 2, both 0.6 m total length, were tracked in May 2008 for 15 and 5 days covering estimated total distances of 150.5 and 98.1 km respectively, prior to the tag becoming detached from fish 1, and a cessation of Argos uplinks from the tag attached to fish 2 (Table 1). Sunfish 3, the largest of the fish tagged (1.0 m TL), was tracked for 92 days between early November 2008 and early February 2009 before the tag stopped transmitting. This fish covered an estimated distance of 1,819 km, moving at a mean speed of 19.8 km d^−1^ compared with movement rates of 10.0 and 19.6 km d^−1^ for sunfish 1 and 2 respectively.

**Figure 2:**
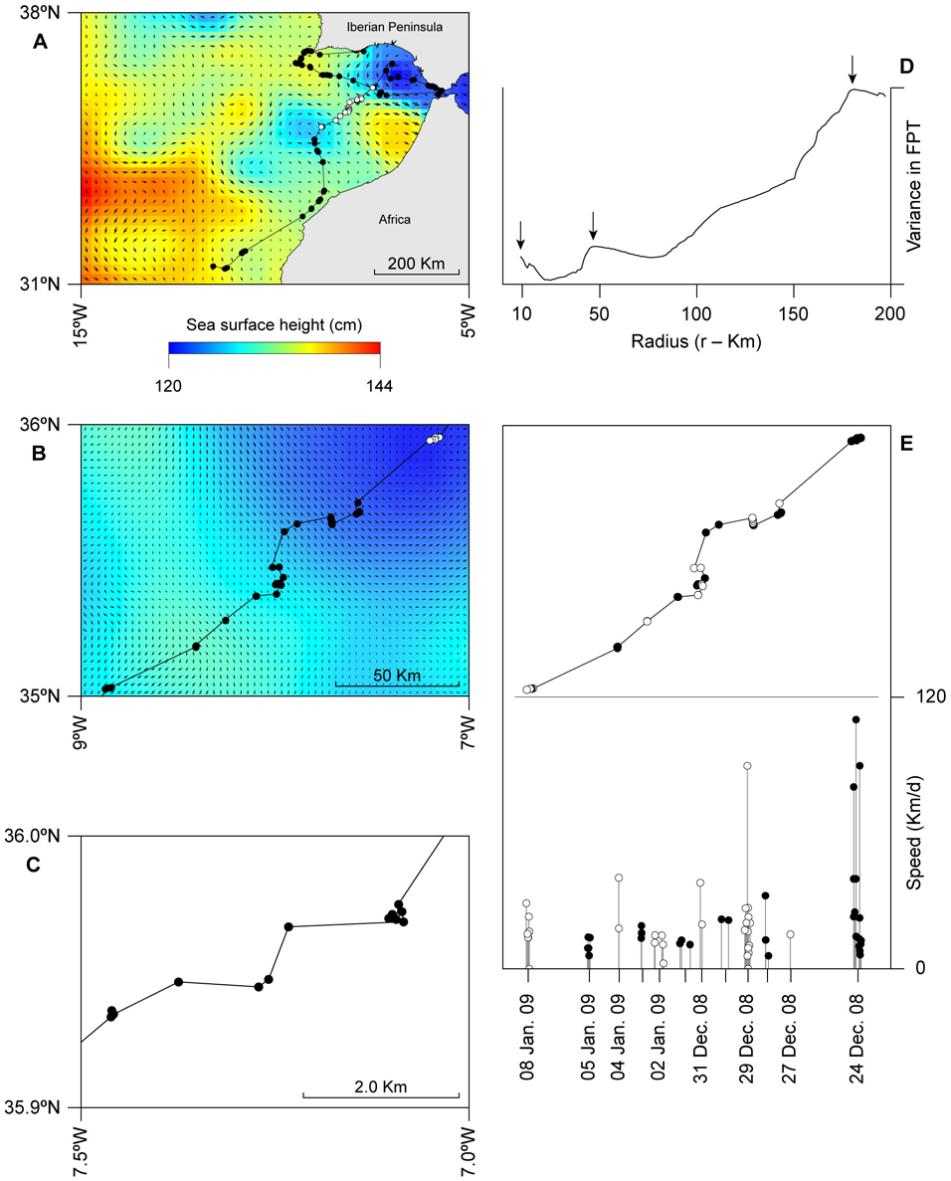
Multi-scale track analysis. (A) Large-scale movement of sunfish S3 in relation to 1/4° altimetry map, depicting averaged mesoscale eddies and geostrophic current direction and speed vectors in the Gulf of Cadiz for the period between 6 November 2008 and 6 February 2009. (B) Track section for the period between 24 December 2008 and 8 January 2009 overlaid on the 1/32° global Naval Research Laboratory Layered Ocean Model (NLOM) SSH data for the same period; white dots in (A) denote the track section shown in (B), and those in (B) are shown in (C) to illustrate the similar patterns in movement at three distinct scales. (D) Variance in first passage times show peaks (arrowed) corresponding to the scales shown in (A–C). (E) Track section illustrating intermittent movement rate over successive days (top panel; white and black circles denote different consecutive days) and variation in over the ground speeds. Minimum time interval between consecutive locations, 4 min.

Filtered Fastloc GPS locations of the path of sunfish 3 over the 3-month period showed it moved west in the days after tagging, prior to heading south-west, then south-eastwards, into the warmer waters of the Gulf of Cadiz as winter progressed (Figure 1C). This fish did not pass through the Strait of Gibraltar when it arrived there, but instead moved north-west into shelf waters south of Cape Trafalgar before heading south-west again across the entire Gulf of Cadiz during January and into cooler, upwelled waters off Morocco prior to moving off-shelf into warmer waters off west Africa in early February (Figure 1C). The Gulf of Cadiz during this period was characterised by several cyclonic and anticyclonic eddies and sunfish 3 generally traversed these features, sometimes going with prevailing geostrophic flow, but at other times moving against and across it (daily data not shown, but see Figure 2A,B for averages). Area restricted movements, evident from consecutive locations occurring close together, were present at the large scale (100 s of km), mesoscale (10 s km) and fine-scales (<2 km) (Figure 2A–C). FPT analysis identified peaks in path variance, signifying transitions from straighter (extensive) movement to more localised (intensive) movement or *vice versa* (Figure 2D), that corresponded to the three distinct spatial scales identified visually (Figure 2A–C).

Analysis of distances between time-stamped GPS locations in a mesoscale section of the track of sunfish 3 between 27 December 2008 and 5 January 2009 (Figure 2E) indicated an intermittent pattern in movement rate. On 27 and 28 December, slower movement rates on each day (<35 km d^−1^) resulted in clustering of consecutive GPS locations, but a faster, directed movement (>90 km d^−1^) between 28 and 29 December relocated the fish to a location 2.8 km away (Figure 2E, lower panel). This pattern of faster, directed movement between area restricted locations was repeated; sunfish 3 moved faster between 30 and 31 December (>35 km d^−1^) before exhibiting slower speeds on 1–3 January, resulting in clustering of locations over these days, before a faster, directed movement on 4 January and slowing again on 5 January (Figure 2E, lower panel). This pattern was not a consequence of drifting with variable speed geostrophic currents since the direction of movement by sunfish 3 was perpendicular to these. The mean distance between locations in these focussed areas was 376.8 m (424.2 S.D., n = 22; position clusters on 28, 29 Dec, 3, 5 Jan).

## Discussion

This study represents the first demonstration of long-term (>90 d) GPS tracking of a large pelagic fish and shows the enormous potential for this technique, where GPS-quality location data is retrieved remotely via conventional Argos satellites. By freeing researchers from the restriction of working on estuarine or nearshore species in order to physically recover tags to download GPS acquisition data [17], this technique presents a whole new capability for tracking large pelagic fish species that surface relatively frequently. The high spatial accuracy of locations this technique yields for fish in open ocean habitats has applications in fisheries and conservation, although it will not be suitable for fish species that remain at depth after tagging. Furthermore, we confirm for this tag type, attachment method and species that higher numbers of more accurate GPS locations were obtained compared with those from Argos PTTs.

The tags we deployed on the two smaller sunfish produced much shorter tracks than anticipated. The tag attached to sunfish 1 became detached after 15 days, whereafter it remained transmitting at the surface for a further week before the batteries were exhausted. It is possible that the relatively large size of the tag and the increased drag from a towed float caused this problem. The cessation of transmissions from the tag attached to sunfish 2 after 5 days may have resulted from tag failure or from the fish dying and sinking to the sea bottom. If it was the death of the fish then we might have expected the tag to re-surface and transmit when it finally broke free from the sunfish carcass (due to scavenger activity). As this did not occur it seems likely that the short tracking time was due to tag failure. Regardless of that contention, it is possible that attaching the tag to a larger sunfish (i.e. S3) resulted in longer term tracking. Watanabe and Sato [18] recorded swimming speeds of three different-sized *Mola mola*, showing that a large (153 kg) individual swam slower (mean and maximum speeds) than two smaller individuals of 48 and 59 kg body mass. In the context of our GPS tracking study, it is conceivable that if relatively slower swimming speeds were exhibited by the larger sunfish (S3), they may have contributed to longer tag retention time through reduction in drag-associated forces acting on the tow body at the point of attachment.

In the early part of the 20^th^ Century contradictory observations about *M. mola* swimming abilities were presented, with them being described as active swimmers in one study [19], and sluggish, inefficient swimmers, passively carried by ocean currents in others [20], [21]. Modern tracking studies of ocean sunfish using attached acoustic transmitters [22], acceleration dataloggers [18] and satellite-linked archival transmitters [16] show sunfish are active swimmers both horizontally and vertically. In this study, GPS tracking of sunfish movements showed them to be active, covering average distances of 10–20 km per day, which is comparable to pelagic shark movement rates [12]. GPS track integration with current direction/strength maps showed sunfish often headed into and across prevailing currents associated with mesoscale eddies. Although sunfish movements have not been considered previously in relation to remotely-sensed ocean current fields, our data nevertheless confirm that *M. mola* are not passive drifters but active swimmers with movement rates within the range observed for pelagic sharks and other pelagic fishes [12], [18].

Self-similar patterns of relatively sharp transitions between area restricted movements and faster, directed movements were apparent at three distinct scales. Particularly at the fine scale, sunfish 3 exhibited pronounced slowing of movement rate over periods of 1–3 days during which movements were spatially constrained with often <500 m between locations during a day; see Results. These apparent ‘stopovers’ in localised areas were interspersed with faster movements on straighter course headings. This interesting insight is as a consequence of having geolocations with low spatial error (between about 26–64 m) relative to the average distances between sunfish re-surfacing locations (e.g. mean ± S.D., 3.3 km ±8.3; *n* = 78 locations, 24 Dec 08 – 8 Jan 09). A similar intermittent pattern of intensive and extensive movement has been observed in filter-feeding basking sharks feeding on patchy zooplankton in shelf waters [23], in foraging leatherback turtles [24] and wandering albatrosses [3], and is reminiscent of birds that stopover to feed and rest during annual migrations [25]. It seems likely that the stopovers shown by sunfish 3 signify encounters with preferred pelagic prey such as gelatinous zooplankton, the distribution of which is highly patchy [26]. Sunfish 3 also moved through thermal frontal areas with relatively sharp horizontal boundaries between cooler, mixed water (14–16°C) and warmer, stratified water (17–18.5°C), particularly when passing along the continental shelf-edge upwelling area off North-west Africa between 17^th^ and 20^th^ January 2009 (see Figure 1C). Ocean sunfish have been observed associated with shelf frontal zones in a previous study [27], although here, sunfish 3 appeared not to remain in these areas for long, for example spending only three days moving on a more or less straight course through the North-west Africa upwelling area, with no apparent ‘stopovers’. These transiting movements by S3 may be a result of low abundance of gelatinous zooplankton prey encountered in that specific region.

The GPS technique we demonstrate for sunfish presents the capability to resolve much finer scale behaviours, such as within and between-patch foraging, than are possible with other techniques presently available for fish tracking. In addition, the technique captures large-scale movements over long periods of time. This suggests that GPS-tracked large pelagic fish could be useful resource detectors of pelagic prey patches or biodiversity ‘hotspots’ in the blue ocean, where satellite remote sensing of ocean colour cannot be used routinely to determine enhanced secondary and tertiary productivity over the appropriate spatio-temporal scales to develop ‘prey fields’ [8]. Furthermore, our results predict the value of long-term GPS tracking applied to other large pelagic fish species such as tunas, billfish and sharks that surface relatively frequently, and have high conservation priority in many ocean regions where a greater understanding of when and why they use certain habitats would enhance management.
